# 930. Bridging Traditions and Technology: Building a Collaborative Burn Care Model with the Amish Community

**DOI:** 10.1093/jbcr/irag033.470

**Published:** 2026-04-06

**Authors:** Kevin Herbert, Anjay Khandelwal, Wayne A Fleck, Richard B Lou

**Affiliations:** Akron Children's Hospital, Akron, OH; Akron Children's Hospital, Akron, OH; Akron Children's Hospital, Akron, OH; Akron Children's Hospital, Akron, OH

## Abstract

**Introduction:**

The Amish are a culturally distinct, medically underserved community whose engagement with the healthcare system presents unique challenges. Prior studies describe recurrent barriers including reliance on home remedies, avoidance of preventive care, mistrust of the “English” medical system, and financial limitations tied to lack of insurance or government assistance. Geographic isolation and transportation barriers further delay access.

To address these issues, our burn center developed a structured, culturally sensitive partnership with the Amish community, integrating communication, education, clinical collaboration, and financial planning.

**Methods:**

In 2020 a structured program was launched to formalize engagement with the Amish including direct communication channels via text/phone, annual conversations, presentations at the Amish National “B & W” Burn Care Meeting, and a training seminar on wound care, therapy, and nutrition built mutual trust and shared knowledge. Pivotal to this was establishing a dual role of Burn Case Manager and Amish Liaison.

Clinically, we integrated Amish caregivers into hospital care by permitting their Dressing Teams to participate in inpatient and outpatient care.

Patient data from a pre-initiative period (2016–2020) was compared with the structured program period (2020–2024). Variables included demographics, burn etiology, surgery rates, ventilator use, length of stay, complications, costs, and discharge disposition.

**Results:**

Implementation of the structured model improved referral patterns and outcomes. Amish patients were transferred earlier from four states, with time to hospital arrival shortened by direct text communication coordinated through the liaison. Over time, the community became increasingly accepting of modern interventions including early excision and grafting, laser scar management, skin cell suspension autografts, and even free tissue transfer. All of these have led to decreased length of stay and decreased complications. The Amish Burn Dressing Team are now integrated into hospital care by working along our team to perform dressing changes utilizing traditional remedies in the burn center.

**Conclusions:**

A structured, culturally sensitive partnership with the Amish community transformed burn care within the region. Central to its success was the role of the case manager as Amish liaison, facilitating communication, coordination, and cultural trust. Compared with outcomes from 2016–2020, the structured program from 2020–2024 achieved earlier referrals, reduced complications, shorter stays, and broader acceptance of modern therapies.

**Applicability of Research to Practice:**

This model illustrates how culturally tailored, liaison-driven collaboration can overcome barriers to specialized care. By merging traditional values with modern innovation, burn centers can created a sustainable, replicable model that advances equity, efficiency, and outcomes in burn care.

**Funding for the study:**

N/A.

